# Hyperosmotic Stress Reduces Melanin Production by Altering Melanosome Formation

**DOI:** 10.1371/journal.pone.0105965

**Published:** 2014-08-29

**Authors:** Bum-Ho Bin, Jinhyuk Bhin, Seung Ha Yang, Dong-Hwa Choi, Kyuhee Park, Dong Wook Shin, Ai-Young Lee, Daehee Hwang, Eun-Gyung Cho, Tae Ryong Lee

**Affiliations:** 1 Bioscience Research Institute, Amorepacific Corporation R&D Center, Yongin, Republic of Korea; 2 Department of Chemical Engineering, POSTECH, Pohang, Republic of Korea; 3 Gyeonggi Bio Center, Gyeonggi Institute of Science & Technology Promotion, Suwon, Republic of Korea; 4 Department of Dermatology, Dongguk University Ilsan Hospital, Goyang, Republic of Korea; Iowa State University, United States of America

## Abstract

Many tissues of the human body encounter hyperosmotic stress. The effect of extracellular osmotic changes on melanin production has not yet been elucidated. In this study, we determined that hyperosmotic stress induced by organic osmolytes results in reduced melanin production in human melanoma MNT-1 cells. Under hyperosmotic stress, few pigmented mature melanosomes were detected, but there was an increase in swollen vacuoles. These vacuoles were stained with an anti-M6PR antibody that recognizes late endosomal components and with anti-TA99 and anti-HMB45 antibodies, implying that melanosome formation was affected by hyperosmotic stress. Electron microscopic analysis revealed that the M6PR-positive swollen vacuoles were multi-layered and contained melanized granules, and they produced melanin when L-DOPA was applied, indicating that these vacuoles were still capable of producing melanin, but the inner conditions were not compatible with melanin production. The vacuolation phenomenon induced by hyperosmotic conditions disappeared with treatment with the PI3K activator 740 Y-P, indicating that the PI3K pathway is affected by hyperosmotic conditions and is responsible for the proper formation and maturation of melanosomes. The microarray analysis showed alterations of the vesicle organization and transport under hyperosmotic stress. Our findings suggest that melanogenesis could be regulated by physiological conditions, such as osmotic pressure.

## Introduction

Many tissues of the human body encounter hyperosmotic stress on a daily basis. For example, renal cells are normally exposed to hyperosmotic stress and maintain the blood osmolality by transporting organic osmolytes [1]–[5]. Sorbitol, inositol, betaine, taurine and glycerophosphorylcholine are known to be organic osmolytes in the kidney [2]. The nucleus pulposus of the intervertebral disc is also confronted with constant osmotic fluctuations based on the composition of the extracellular matrixes, including mechanical forces [6], [7]. In the skin, water transport and cell hydration induce osmotic stress [8], [9]. Keratinocytes maintain cell volume homeostasis by regulating the expression of organic osmolyte transporters [10], [11]. Because organic osmolytes are involved in antioxidant defenses, protein stabilization and stress responses, human dermal fibroblasts sometimes express organic osmolyte transporters, and osmolytes accumulate inside the cells [12].

When cells encounter hyperosmotic stress, the biogenesis of intracellular cargo and lysosomal and endocytic compartments is significantly affected [13], [14]. The presence of excessive organic osmolytes within cells induces the swelling of mannose 6-phosphate receptor (M6PR)-positive late endosomes [13], which occurs as a consequence of endocytic membrane influx coupled with the failure to transport cargo from the membrane to other intracellular destinations [14].

The melanosome is a lysosome-related organelle and is a dynamic place to synthesize melanin. To produce melanin, the melanosome should be properly matured from Stage I to IV in a step-wise manner, and this maturation is largely dependent on organelle formation and the transport of melanogenesis-related proteins [15]. Hermansky-Pudlak syndrome (HPS) is an autosomal recessive disorder that affects the biogenesis and transport of intracellular cargo and transport systems, especially lysosome-related organelles, such as platelet dense granules and melanosomes [16], [17]. Patients with this disorder demonstrate bleeding and cellular storage disorders or hypo-pigmentation [18]–[20] because of the mutations on the genes involved in the biogenesis of lysosome-related organelle complexes (BLOCs) and adaptor protein complexes (APs), which are crucial for organelle biogenesis associated with melanosomes, platelet dense granules, and lysosomes [21], [22], indicating that the regulation of melanogenesis could be considered an aspect of organelle formation and cargo transport.

Because the skin is an organ that is regulated by osmotic stress [8]–[12] and because melanosomes are lysosome-related organelles and their biogenesis could be affected by hyperosmotic stress, it is possible that melanogenesis is regulated by changes in osmotic pressure. In this study, we examined the osmotic effects on melanosome formation and melanin production in human melanoma MNT-1 cells, which are highly pigmented and include well-organized mature melanosomes of stage III and IV. We found that melanin production was remarkably reduced when osmotic stress was applied and showed that this phenomenon resulted from defects in melanosome formation. Finally, we showed that the vesicle organization and transport are altered in hyperosmotic conditions using a microarray analysis, and a dysfunctional PI3K pathway is involved in these steps.

## Results

### Hyperosmotic stress reduces melanin production

**Figure 1:**
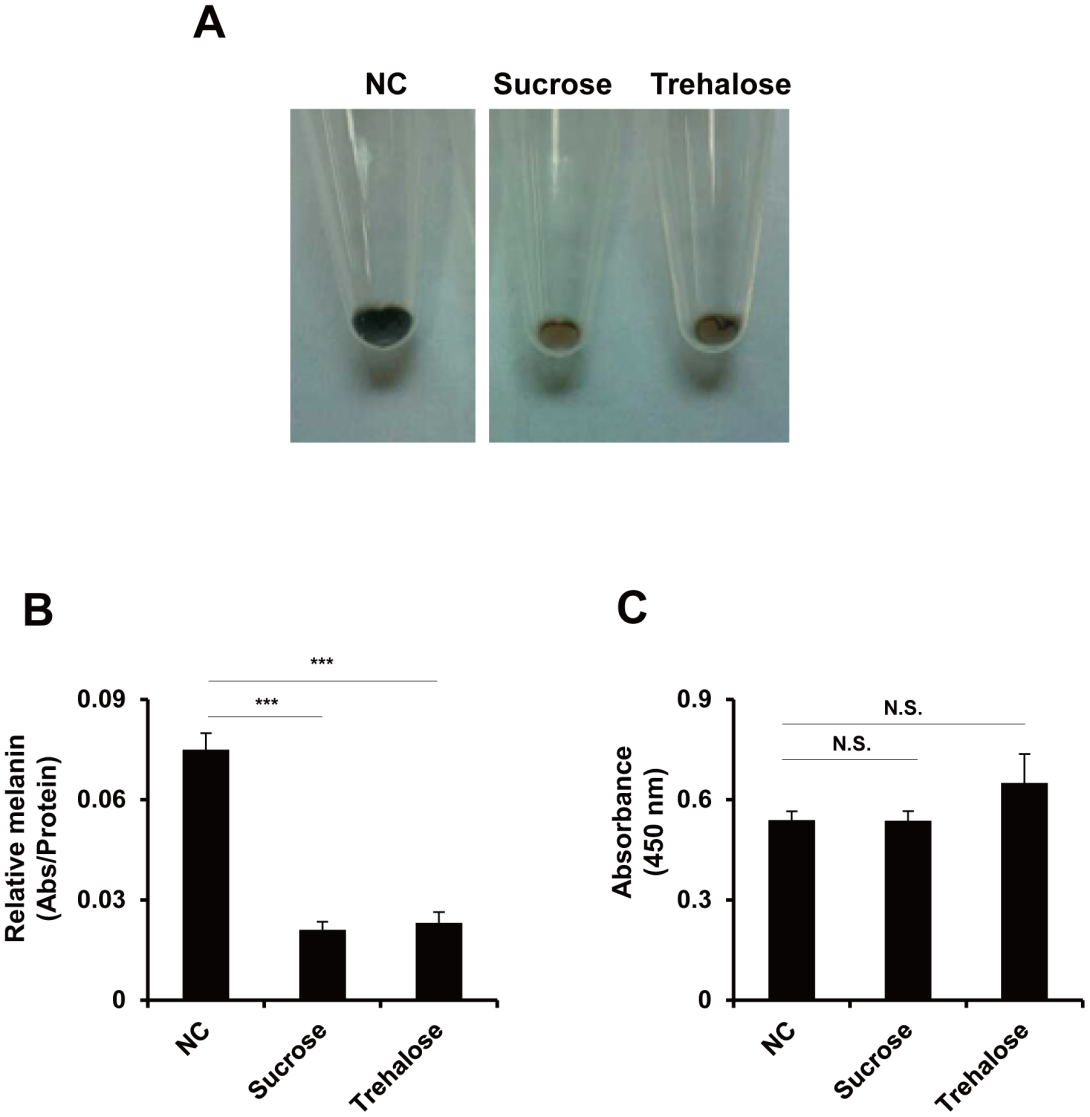
Hyperosmotic stress down-regulates melanin production. Hyperosmotic stress reduces melanin production but does not affect tyrosinase activity in MNT-1 cells. (A) MNT-1 cells were treated with 50 mM of the indicated sugar for 7 days, and the color of the cell pellets was monitored. NC, negative control. (B) The melanin content was measured at 450 nm. The data are representative of three independent experiments (***, P < 0.005). (C) Tyrosinase activity assay. MNT-1 cell extracts were incubated with 10 mM L-dihydroxyphenylalanine (L-DOPA), and the amount of melanin was measured at 450 nm. The data are representative of three independent experiments (N.S., not significant).

To elucidate the effect of osmolyte overloading on pigmentation, we treated highly pigmented human melanoma MNT-1 cells with sucrose or trehalose as osmolytes. After 7 days of treatment with disaccharides, we found a remarkable reduction in the melanin production: 71.8 ± 3.2% with sucrose treatment and 69.2 ± 4.4% with trehalose treatment compared with the control (Figure 1A, B). The tyrosinase activity remained unchanged (Figure 1C), suggesting that an alternative pathway is involved in hypo-pigmentation by hyperosmotic stress.

### Hyperosmotic stress induces the formation of M6PR-positive swollen vacuoles that contain TYRP-1 and PMEL17

**Figure 2:**
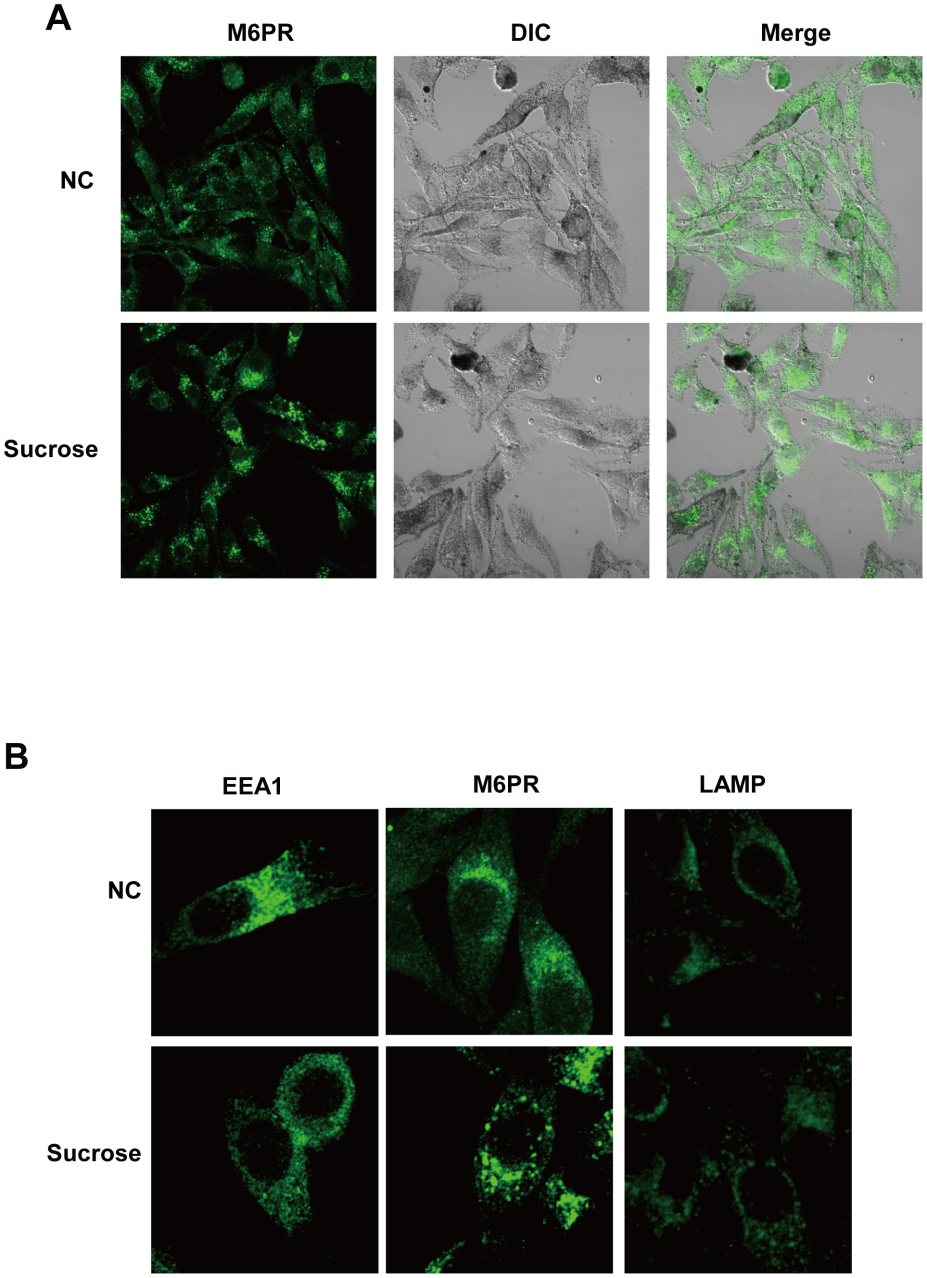
Hyperosmotic stress induces vacuolation. Hyperosmotic stress induces swelling of M6PR-positive vacuoles. (A) MNT-1 cells were treated with 50 mM sucrose for 12 hours. After fixation in 3% paraformaldehyde and permeabilization with 0.1% Triton X-100 in phosphate-buffered saline (PBS), the cells were stained with an anti-M6PR antibody and examined by confocal microscopy at a magnification of 400×. NC, negative control; DIC, differential interference contrast. (B) MNT-1 cells were treated with 50 mM sucrose for 7 days and stained with the intracellular vesicle markers anti-EEA1, anti-M6PR or anti-LAMP. The images were acquired using a confocal microscope at 1260×.

The biogenesis and transport of endosomes and lysosomes are affected by hyperosmotic stress [13], [14] and these compartments are related to melanosome formation [15], [21]. We examined the state of endosomes and lysosomes under hyperosmotic conditions. Human melanoma MNT-1 cells were treated with 50 mM sucrose, and we analyzed the morphology of the intracellular organelles by immunofluorescence staining with the following antibodies: early endosome antigen 1 (EEA1) for early endosomes, M6PR for late endosomes and lysosomal membrane protein 1 (LAMP1) for lysosomes. We found that sucrose treatment induced M6PR-positive puncta in the majority of cells within 12 hours after treatment (Figure 2A); this phenomenon was sustained for 7 days (Figure 2B, M6PR). The signals from the anti-EEA1 and anti-LAMP antibodies were comparable between the control cells and the sucrose-treated cells (Figure 2B, EEA1; LAMP).

**Figure 3:**
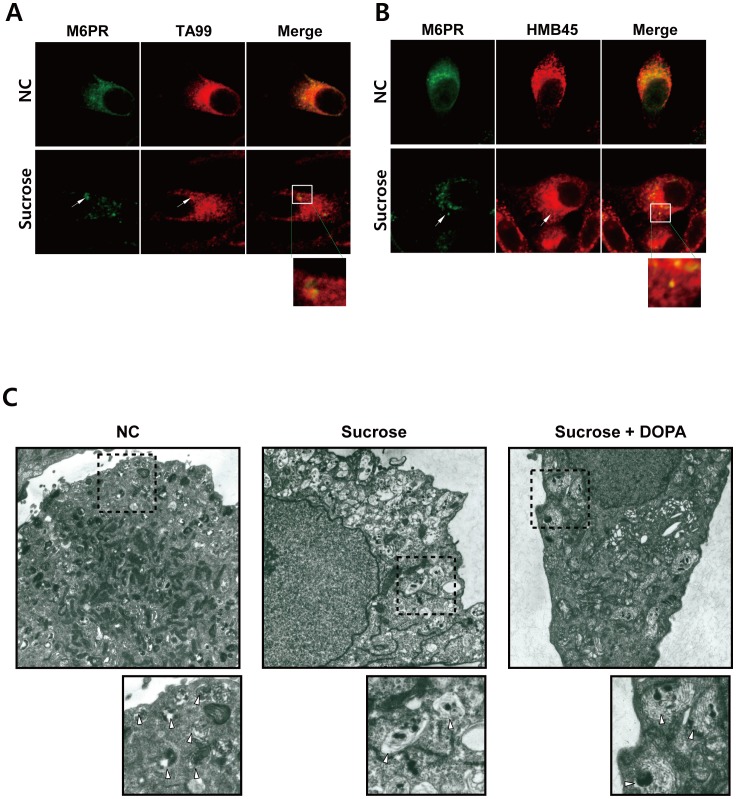
Hyperosmotic stress-induced M6PR-positive swollen vacuoles contain TYRP-1 and PMEL17. MNT-1 cells were treated with 50 mM sucrose for 7 days and stained with anti-TA99 (A) or anti-HMB45 (B) antibodies. The fluorescence images were acquired using confocal microscopy at a magnification of 1260×. The insets show the magnified images. NC, negative control. (C) Electron microscopic analyses were performed on MNT-1 cells before and after 50 mM sucrose treatment for 7 days. For the in situ L-DOPA assay, the MNT-1 cells were incubated with 0.1% L-DOPA for 3 hours after treatment with 50 mM sucrose for 7 days (Sucrose + DOPA). The insets show the magnified images. The arrowheads indicate various stages of melanosomes (NC), swollen vesicles containing dark granules (Sucrose), or swollen vesicles after the in situ DOPA assay (Sucrose + DOPA). The dotted lines identify the position for the magnified images. NC, negative control.

To determine whether these M6PR-positive puncta were related to melanosomes, we examined the extent of M6PR colocalization with the melanosome-associated proteins TA99 (TYRP-1) or HMB45 (PMEL17), which are well-known melanosomal marker proteins [23]. The M6PR-positive swollen vacuoles overlapped with the expression of TYRP-1 and PMEL17 (Figure 3A, B, inset), implying that these vacuoles could be defined as melanosomes. On the electron microscopic analysis, the various stages of melanosomes were observed in the untreated control cells (Figure 3C, NC, inset, arrowheads). However, the sucrose-treated cells contained many enlarged vacuoles (Figure 3C, Sucrose, inset, arrowheads) that contained dark melanized granules. These melanized granules were spotted and were not dispersed along the melanosomal matrix. The faintly detected melanosomal matrices in the swollen vacuoles were more clearly detected after L-DOPA incubation (Figure 3C, Sucrose+DOPA), suggesting that the vacuoles were still capable of melanin synthesis. The expression of melanogenesis-related proteins, such as TYR, TYRP-1, MART-1, and PMEL17, was not significantly affected by hyperosmotic stress (Figure S1A in File S1). The processing and maturation of the major melanosomal matrix protein PMEL17 were normal under hyperosmotic stress (Figure S1B, C in File S1), indicating that there were no apparent defects in the expression level of the melanogenesis-related proteins. The environment inside the swollen vacuoles was more likely to be incompatible with melanin synthesis, resulting in less melanin contents. In addition to swollen vacuoles, we observed that in sucrose-treated cells, the merged expressions of M6PR with TYRP-1 or PMEL17 were apparently reduced compared to negative control (Figure 3A, B), implying that hyperosmotic stress disturbs the proper vesicle trafficking for melanosome formation and this might be also involved in the hypo-pigmentation.

### The PI3K pathway is crucial for melanosome formation

**Figure 4:**
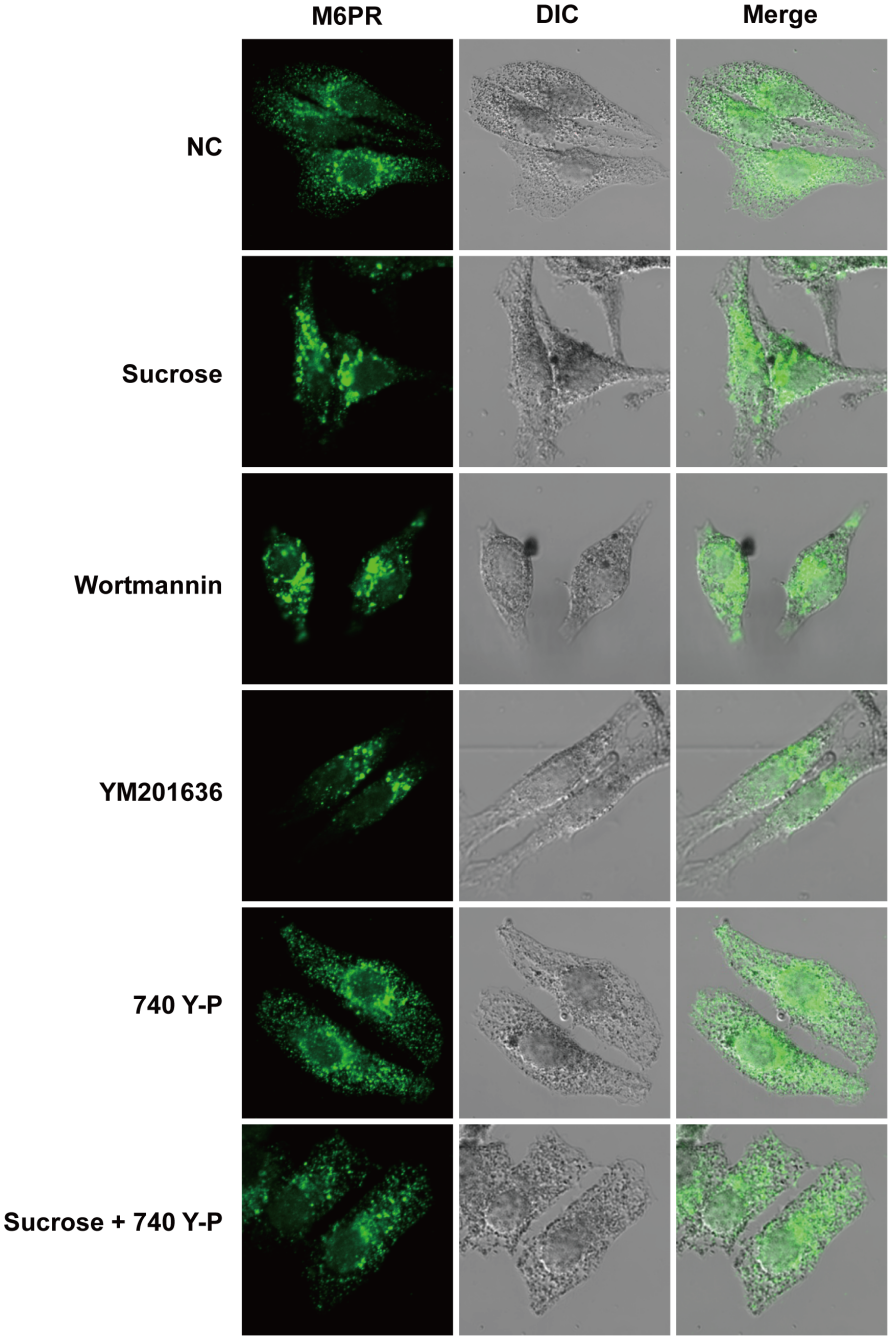
PI3K activation inhibits hyperosmotic stress-induced vacuolation. MNT-1 cells were treated with 50 mM sucrose, 10 µM wortmannin, 10 µM YM201636, or 20 µM 740 Y-P for 24 hours. After fixation in 3% paraformaldehyde and permeabilization with 0.1% Triton X-100 in phosphate-buffered saline (PBS), the cells were stained with an antibody against M6PR and examined using confocal microscopy at a magnification of 1260×. NC, negative control; DIC, differential interference contrast.

PI3K inhibitors induce the swelling of M6PR-positive late endosomes and block the fusion of dense core lysosomes with late endosomes in normal rat kidney epithelial (NRK) cells [13], [14]. We treated human melanoma MNT-1 cells with the PI3K pathway inhibitors wortmannin and YM201636 for 24 hours, and we stained for M6PR. Both inhibitors induced the formation of M6PR-positive puncta similar to the sucrose-induced vacuoles (Figure 4, Sucrose; Wortmannin; YM201636). We examined the effect of the PI3K activator 740 Y-P. The results showed that 740 Y-P alone did not cause any changes (Figure 4, 740 Y-P), but it remarkably reduced the number of M6PR-positive vacuoles induced by the sucrose treatment (Figure 4, Sucrose+740 Y-P), suggesting that inhibition of the PI3K pathway is involved in sucrose-induced vacuolation. These data indicate that the PI3K pathway plays a critical role in the vesicle trafficking that is required for melanosome formation.

### Genome-wide analysis of gene expression under hyperosmotic stress

**Figure 5:**
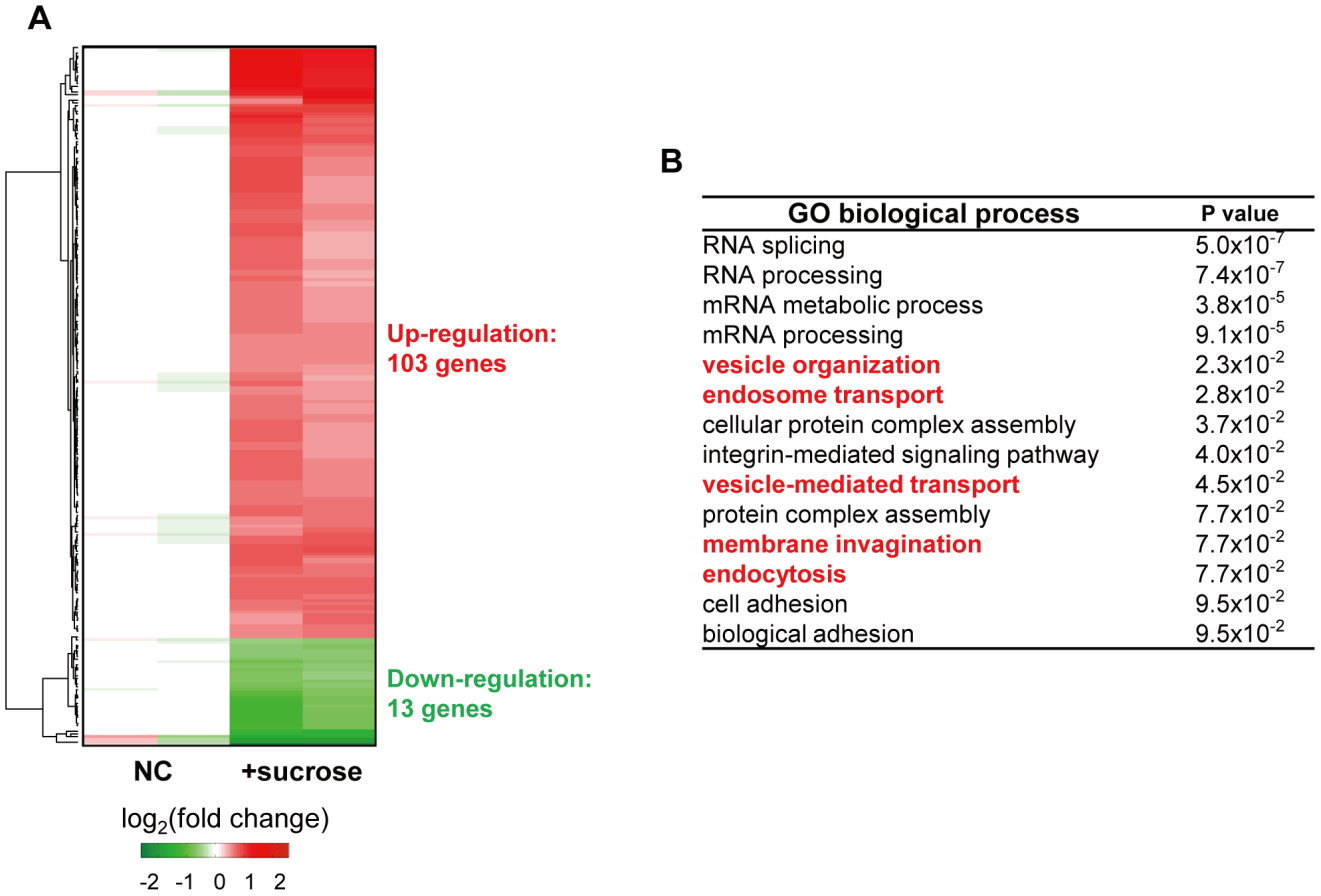
Genome-wide analysis reveals that hyperosmotic stress affects the expression of genes involved in intracellular vesicle transport. (A) log_2_-fold-changes of up-regulated (red) or down-regulated (green) genes were displayed after hyperosmotic stress by sucrose treatment. The cells were treated with 50 mM sucrose for 7 days, and the microarray analysis was performed using 10 µg of the total RNA. The color bar represents gradients of log2-fold-changes in each comparison. NC, negative control. (B) The biological processes that were significantly up-regulated in sucrose-treated cells were summarized after gene ontology (GO) analysis.

To identify the genes that were influenced by hyperosmotic stress, a genome-wide analysis of gene expression was performed and systemically analyzed (Figure 5). Under hyperosmotic stress, 103 genes were up-regulated (Figure 5A and Table S1). Based on the functional enrichment analysis using DAVID [24], many of these genes were involved in vesicle transport, including vesicle organization, endosome transport, vesicle-mediated transport, membrane invasion and endocytosis (Figure 5B). We further verified the enrichment of specific representative genes involved in vesicle transport, EEA1, LYST, ZFYVE16 and TFRC (Figure S2A in File S1). To examine if some of those are involved in the vesicle organization and transport under hyperosmotic stress and have the impact on the decrease in melanin production, we performed the knockdown experiment using siRNAs against EEA1 or LYST in the presence of sucrose, but could not observe any recovery effect by simply deleting these genes (Figure S2B in File S1). These results indicate that hyperosmotic stress induces broad changes in gene expression, especially in the expression of genes involved in vesicle trafficking, and this may induce disturbances on vesicle-mediated transport involved in melanosome formation, resulting in reduced melanin synthesis.

## Discussion

**Figure 6:**
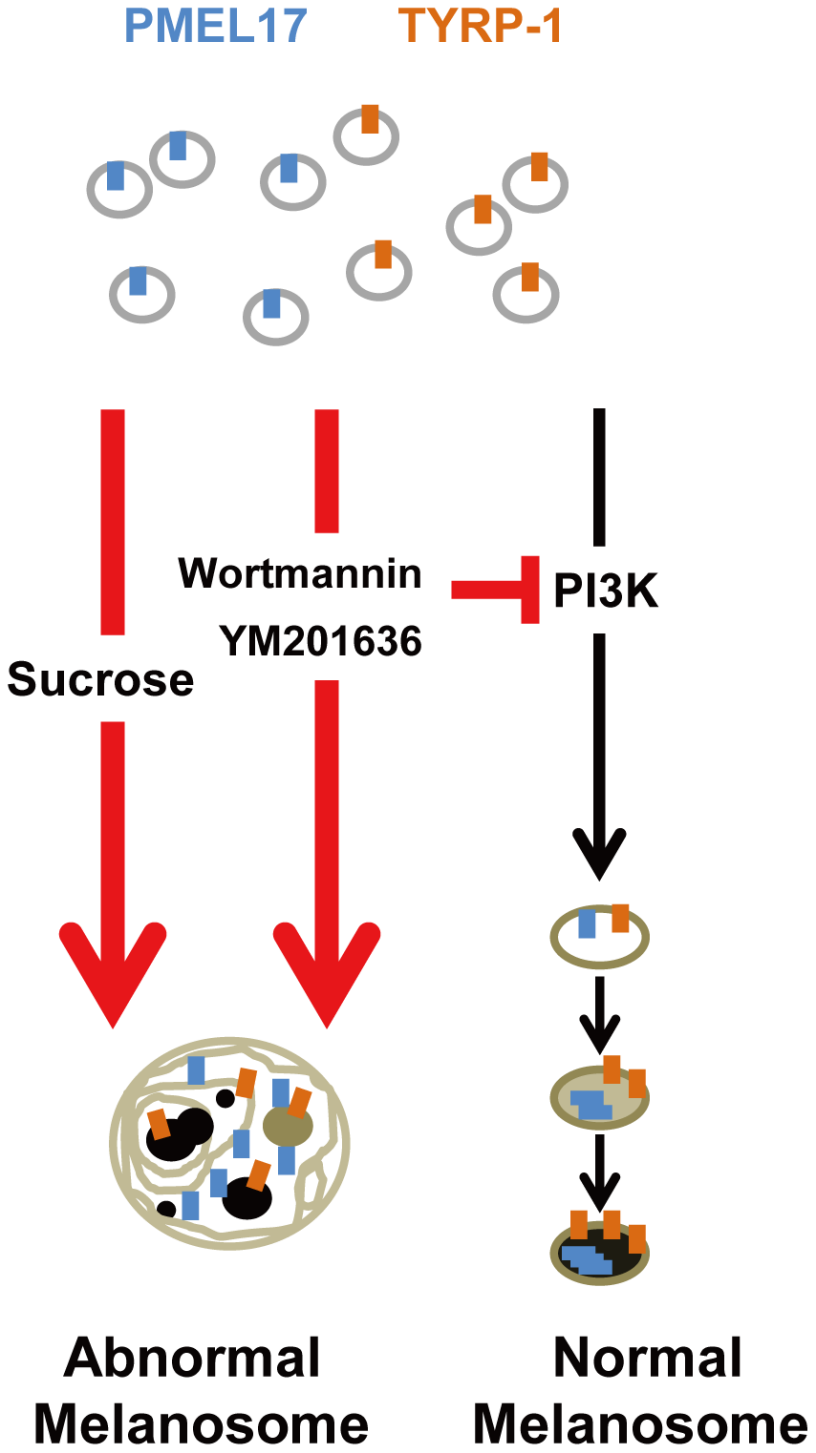
Schematic model of hypo-pigmentation under hyperosmotic stress. Hyperosmotic stress induces swollen, abnormal melanosomes in which the inner condition is not appropriate for melanin production. The PI3K pathway is involved in proper vesicle trafficking for melanosome formation and maturation. Thus, disruption of this pathway by sucrose-induced hyperosmotic stress results in the formation of swollen, abnormal melanosomes.

In this study, we observed the down-regulation of melanin production under hyperosmotic stress in human melanoma MNT-1 cells. In our model, hyperosmotic stress disrupts proper vesicle trafficking and inhibits proper melanosome formation, resulting in the formation of swollen vacuoles and in reduced melanin synthesis (Figure 6). We confirmed that hyperosmotic stress had the same effect on normal human melanocytes by demonstrating swollen M6PR-positive vacuoles and reduced melanin production (Figure S3 in File S1).

The skin is an organ that experiences osmotic changes. In previous reports, keratinocytes and fibroblasts were shown to express specific proteins and transporters to maintain the cellular homeostasis under hyperosmotic conditions [10]–[12]. However, there are no reports of the hyperosmotic effects on melanotic cells. Because melanotic cells have melanosomes that are lysosome-related organelles [13], [14], we expected that hyperosmotic stress could affect melanogenesis. When human melanoma MNT-1 cells and melanocytes were treated with sucrose, we observed the decreased melanin content, the reduced merged expression of M6PR with TYRP-1 or PMEL17 and swollen vacuoles, implying that hyperosmotic stress disturbs the proper vesicle trafficking for melanosome formation. Genome-wide analysis of gene expression identified about one hundred molecules influenced by hyperosmotic stress, and major alteration was observed in genes related to vesicle transport. The treatment of siRNAs targeting the representative EEA1 or LYST among up-regulated genes did not recover the melanin reduction caused by hyperosmotic stress (Figure S2B in File S1), suggesting that selected or single gene might be not enough to induce the malformation of melanosome under hyperosmotic condition. Or, the alteration of gene expression may be a result of cellular adaptation to the changes in vesicle organization and transport under hyperosmotic stress.

Melanogenesis-related proteins are transported to the melanosome via vesicle-sorting mechanisms [21], [25], [26]. PMEL17 is sorted into early endosomes and deposited into early stage melanosomes [25]. TYRP-1 is sorted into late-stage melanosomes [21], [25]. Our data showed that hyperosmotic stress induces M6PR-positve swollen vacuoles, which are positive for both HMB45 and TA99 (Figure 3). Because the anti-HMB45 antibody detects PMEL17 in early-stage melanosomes [23], [27], and given that the anti-TA99 antibody recognizes TYRP-1 in late-stage melanosomes [25], [28] and that M6PR is normally used as a marker for late-stage endosomes [29], [30], these vacuoles seemed abnormal, and the important step for melanosome formation was stalled, resulting in a halt in the proper maturation and reduced melanogenesis. An *in situ* L-DOPA assay demonstrated that the swollen vacuoles could still synthesize melanin (Figure 3C), and the expression/modification/processing of melanogenesis-related proteins was not changed under hyperosmotic conditions (Figure S1 in File S1), implying that the necessary components for melanin synthesis are present but the vacuole inner condition is not appropriate for melanin production.

We showed that hyperosmotic stress caused by organic osmolytes induced the swelling of M6PR-positive vacuoles, as was previously reported [13], [14]. Organic osmolytes such as sucrose are hydrophilic and might not permeate a cell membrane spontaneously. These osmolytes should be transported into cells via transporters or via endocytosis. If the organic osmolytes are present in excessive amounts, they could enter the cells via endocytosis and accumulate within endocytic compartments, which are generally destined for late endosomes, triggering hyperosmotic stress within the lumen of the endocytic compartments. As a consequence of hyperosmotic conditions within the lumen, water enters the lumen, and the swollen structure might appear. Our data showed that EEA1-positive early endosomes do not appear to be swollen (Figure 2B), which might be the reason that the early endosome converts to the late endosome relatively fast before swelling occurs. The use of labeled osmolytes could provide more precise information regarding the localization of osmolytes and the affected vesicle types, which remains to be further examined.

The immunofluorescence analysis revealed that hyperosmotic stress induces two distinct types of vacuoles, M6PR-positive abnormal melanosomes or LC3-positive vacuoles that are possible autophagosomes (Figure S5 in File S1). When cells encounter hyperosmotic stress, they have been reported to activate autophagy and promote microtubule-dependent autophagosomal clusters, resulting in the alteration of cellular components, adaptation to hyperosmotic conditions, and improvements in survival [31]. We examined if melanotic cells activate autophagosome formation under hyperosmotic conditions by detecting LC3, an autophagosome marker [32]. MNT-1 cells activate LC3 under sucrose-induced hyperosmotic conditions (Figure S4A and B in File S1). However, we could not detect any changes of the expression level of melanogenesis-related proteins, which are expected in cases of autophagosome activation (Figure S1 and S4C in File S1); these results indicate that hyperosmotic stress is not involved in the degradation of melanogenesis-related proteins in our model. The microarray data from the MNT-1 cells after exposure to hyperosmotic stress for 7 days did not reveal any alterations in the expression of autophagy-related genes (Table S1), suggesting that autophagy does not occur in this condition. However, how hyperosmotic stress activates LC3 and whether the activated autophagy might promote the degradation of some other proteins that are essential for melanosome formation remains unclear. Detailed analyses using specific suppression of autophagic factors such as ATG5 or ATG7 or autophagy inducers in pigment cells such as APR101 might facilitate the elucidation of these questions, as was previously reported [33], [34]. Previously, trehalose, but not sucrose, was reported to induce autophagy via an mTOR-independent mechanism [35]. We do not exclude the possibility that the hypo-pigmentation mechanism might be different according to the type of organic osmolytes. However, various disaccharides, including sucrose and trehalose, induced hypo-pigmentation (Figure S6 in File S1), implying that our proposed hypo-pigmentation mechanism could be widely adapted.

We showed the effects of hyperosmotic stress on the reduction of melanin production in human melanotic cells by inducing abnormally swollen melanosomes. Our data propose possible new mechanisms of the alteration of melanin production by physiological changes of osmotic conditions and provide new insights on melanosome dynamics, including melanosome maturation and melanogenesis-related protein transport, under physiological environments.

## Materials and Methods

### Cell culture and materials

The human melanoma MNT-1 cell line was cultured as previously described with minor modifications [36]. Briefly, the cells were maintained at 37°C in MEM (Gibco, Carlsbad, CA, USA) with 20% fetal bovine serum (Gibco). Wortmannin (Cell signaling, Danvers, MA, USA), YM201636 (Cell signaling), 740 Y-P (R&D systems, Minneapolis, MN, USA) and Cyclohexamide (Sigma, St. Louis, MO, USA) were purchased and dissolved in DMSO to produce stock solutions. Sucrose, trehalose, cellobiose, maltose, lactose and lactulose (all from Sigma) were purchased and dissolved in culture medium and sterilized by filtration with a 0.45-μm pore size filter before use. Designed siRNA mixtures (Genolution, Seoul, Korea) and Lipofectamine RNAimax (Invitrogen, CA, USA) were purchased and treated as the manufacturer describes.

### Melanin and tyrosinase activity assay

Approximately 1 × 10^5^ cells were collected and centrifuged at 500 ×g for 5 minutes. The cell pellets were washed with PBS and dissolved in 1 N NaOH for 1 hour. The melanin levels were determined by measuring the absorbance at 450 nm. For the tyrosinase activity assay, the cells were lysed with 1% NP-40 in PBS and clarified by centrifugation for 20 minutes at 16,000 ×g. L-DOPA in PBS (2 mg/ml) was added to each lysate and incubated for 1 hour. The absorbance was measured at 450 nm.

### Western blot analysis and fluorescence microscopy

The cells were lysed with 1% NP-40 in a solution of 0.05 M Tris-HCl, pH 7.5, 0.15 M NaCl, 0.01 M MgCl_2_. The cell debris was removed by centrifugation at 16,000 ×g for 20 minutes. The supernatants were removed, and the protein content was quantified using a BCA assay. An aliquot of 20 µg of protein was loaded into each well of an SDS-PAGE gel. For immunoblotting, the SDS-PAGE gel was electroblotted onto a PVDF membrane. An anti-TYRP-1 antibody (Santa Cruz Biotechnology, Santa Cruz, CA, USA), anti-GAPDH antibody (Santa Cruz Biotechnology), anti-MART-1 antibody (Thermo Fisher Scientific, CA, USA), anti-HMB45 antibody (GeneTex, Irvine, CA, USA), anti-TA99 antibody (GeneTex), anti-PMEL17 (aN) (GeneTex) and anti-TYR antibody (Upstate Biotechnology, Lake Placid, NY, USA) were used for protein detection. For the fluorescence microscopy, cells were cultured on Lab-Tek chamber slides (Nunc, NY, USA), fixed with 4% paraformaldehyde in PBS, permeabilized with 0.1% Triton X-100 in PBS containing 1% BSA for 5 minutes, and incubated with a primary antibody. Fluorescence was detected by secondary antibody staining with the Alexa Fluor 488-conjugated F(ab')2 fragment of goat anti-mouse IgG (Invitrogen) and the Alexa Fluor 594-conjugated F(ab')2 fragment of goat anti-rabbit IgG (Invitrogen).

### Transmission Electron Microscopy

The cells were fixed using Karnovsky fixative for 30 min at RT, collected by centrifugation and embedded in a low-melting-point agarose matrix. The agarose-embedded cells were post-fixed in 2% osmium tetroxide and stained with uranyl acetate. The specimens were dehydrated through a graded ethanol series and embedded in Embed-812 (Electron Microscopy Sciences, Hatfield, PA) at 60°C for 48 hours. The sections (approximately 70-90 nm) were stained using uranyl acetate and lead citrate and observed using a transmission electron microscope (TEM; JEM-1200 EX, JEOL).

### Quantitative real-time PCR

The total RNA was isolated using trizol (Invitrogen) and was reverse-transcribed into cDNA using ReverTra Ace (Toyobo, Osaka, Japan) according to the manufacturer's instructions. The gene expression analysis was performed using TaqMan Universal Master Mix and TaqMan Gene Expression Assays (Applied Biosystems, Foster City, CA, USA) according to the manufacturer's instruction.

### Microarray analysis

Duplicate experiments were performed on MNT-1 cells before and after sucrose treatments for 7 days. In each experiment, the total RNA was isolated from the MNT-1 cells that had been independently cultured using trizole (Invitrogen). The total RNA was reverse-transcribed, amplified, and hybridized onto an Agilent Human GE 4x44K V2 chip according to the manufacturer's protocol. The array was scanned using the Agilent G2565CA Microarray Scanner. We normalized the log2-intensities of all of the probes using the quantile normalization method [37]. The expression of each probe was determined using a Gaussian mixture distribution for the log2-intensities as previously described [38]. We identified the differentially expressed genes (DEGs) from the comparisons of before versus after sucrose treatment using the previously reported integrative statistical hypothesis testing method [39] that combines the adjusted P-value (*Pad*) from the two-tailed t-test and the median ratio test. We filtered the probes using P-values greater than 0.1 (*Pt*) from the two-tailed t-test to capture false positives caused by fluctuations between the replicates. The DEGs under hyperosmotic stress were identified as the genes with *Pad*<0.01, *Pt*<0.1, and fold-changes≥1.5 among the expressed genes. Functional enrichment analysis for the up-regulated genes was performed using DAVID Bioinformatics Resources [24] and Gene Ontology (GO) biological processes; processes with P-values<0.1 were considered statistically significant. Microarray data can be obtained from NCBI Gene Expression Omnibus (GEO), accession: GSE57565.

### Statistical analysis

A two-tailed Student's t test was used to analyze the differences between the two groups.

## Supporting Information

File S1 **Figure S1. The expression and processing of melanogenesis-related proteins is normal under hyperosmotic stress.** (A) MNT-1 cells were treated with 50 mM sucrose for 7 days, and the expression levels of melanogenesis-related proteins were analyzed by western blotting using the indicated antibody. NC, negative control. (B) The recognition sites of PMEL17 by aN or an anti-HMB45 antibody are schematically represented. aN, anti-PMEL17 antibody that recognizes the N-terminal region of PMEL17, as indicated. (C) The processing of PMEL17 in MNT-1 cells treated with 50 mM sucrose for 7 days was analyzed by western blotting. NC, negative control. **Figure S2. The expression of vesicle transport-related transcripts.** (A) Non-treated (NC) or MNT-1 cells treated with 50 mM sucrose for 7 days were harvested. The expression levels of vesicle transport-related transcripts that were up-regulated under hyperosmotic stress in the microarray data (Table S1) were quantified using RT-qPCR. The data are representative of three independent experiments (***, *P* < 0.005). (B) Each siRNA was treated twice every third day during sucrose treatment for 7 days, and the color of the cell pellets was monitored. NC, negative control. **Figure S3. Hyperosmotic stress down-regulates melanin production in normal human melanocytes by inducing abnormal, swollen melanosomes.** (A) Normal human melanocytes were treated with 50 mM for 7 days, and the color of the cell pellets was monitored. NC, negative control. (B) The melanin content was measured at 450 nm. The data are representative of three independent experiments (***, P < 0.005). (C) Hyperosmotic stress-induced M6PR-positive swollen vacuoles contain TYRP-1 and PMEL17 in normal human melanocytes. Cells were treated with 50 mM sucrose for 24 hours and stained with anti-TA99 or anti-HMB45 antibodies. The fluorescence images were acquired using confocal microscopy at a magnification of 1260×. NC, negative control. **Figure S4. Hyperosmotic stress induces LC3-positive vacuoles that are distinct from M6PR-positive vacuoles.** MNT-1 cells were treated with 50 mM sucrose for 7 days and stained with anti-LC3, anti-M6PR or anti-HMB45 antibodies. The fluorescence images were acquired using confocal microscopy at a magnification of 1260×. Some LC3-positive vacuoles are positive for HMB45 (Lower). The arrowheads indicate the colocalization of LC3 and HMB45. The images were analyzed using confocal microscopy at 1260×. **Figure S5. Hyperosmotic stress activates LC3 but does not improve the degradation of melanogenesis-related proteins.** (A) MNT-1 cells were treated with 50 mM sucrose for 24 hours, and the expression level of LC3 was analyzed by western blotting using the anti-LC3 antibody. (B) MNT-1 cells were treated with 50 mM sucrose for each indicated time period, and the expression level of LC3 was analyzed by western blotting using the anti-LC3 antibody. (C) MNT-1 cells were treated with 50 mM sucrose for each indicated period in the presence of Cycloheximide (CHX), and the expression levels of the melanogenesis-related proteins were analyzed by western blotting using each indicated antibody. Each band was analyzed using imageJ software (http://rsbweb.nih.gov/ij/download.html). (D) MNT-1 cells were treated with 50 mM sucrose for 24 hours, and the expression levels of melanogenesis-related proteins were analyzed by western blotting using each indicated antibody. **Figure S6. The hypo-pigmentation effect of various disaccharides.** (A) MNT-1 cells were treated with 50 mM of the indicated sugar for 5 days, and the color of the cell pellets was monitored. NC, negative control. (B) The melanin content was measured at 450 nm. The data are representative of three independent experiments (**, P < 0.01, and ***, P < 0.005).
(PDF)

Table S1 List of up- or down-regulated genes under hyperosmotic stress. The raw intensity values (before and after sucrose treatment), log2-fold-changes, and adjusted P-values (Pad) computed from two-tailed t-tests and median ratio tests (see Materials and Methods) are given. The up- or down-regulated genes are represented by each ‘up’ or ‘down’ in the table.
(PDF)
