# Supplementary figures and images for: Hyperosmotic Stress Reduces Melanin Production by Altering Melanosome Formation
Source: PLoS One. 2014 Aug 29;9(8):e105965. doi: 10.1371/journal.pone.0105965 (PMC4149489; doi:10.1371/journal.pone.0105965)

**A**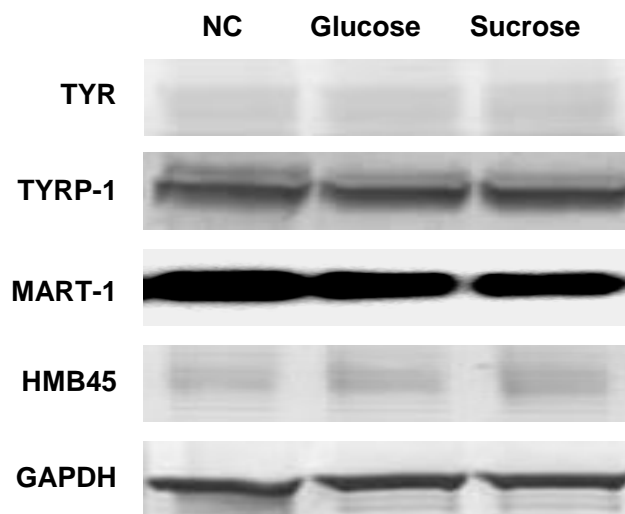**B**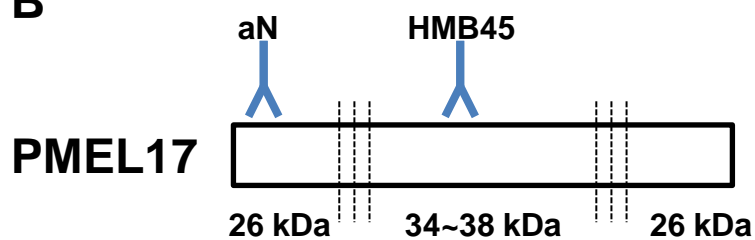**C**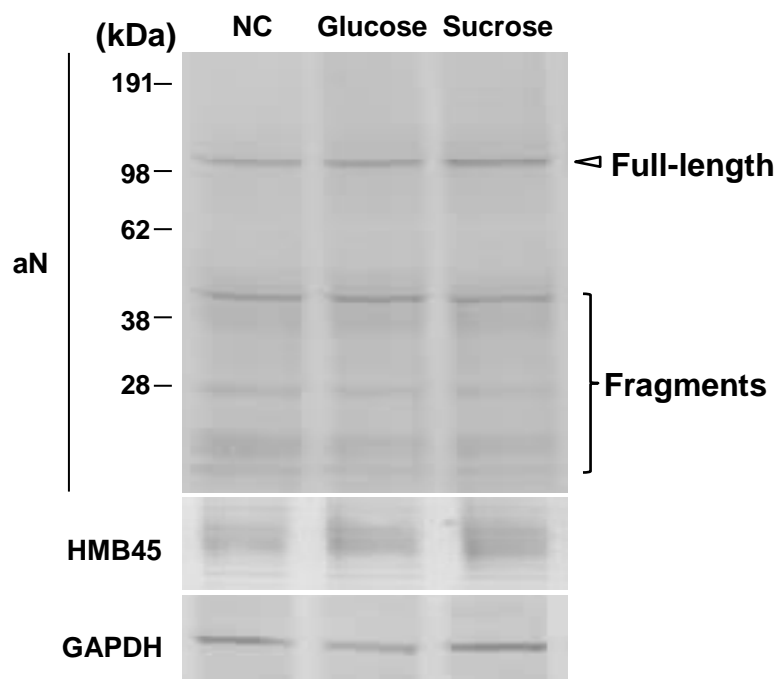**Figure S1**

**A**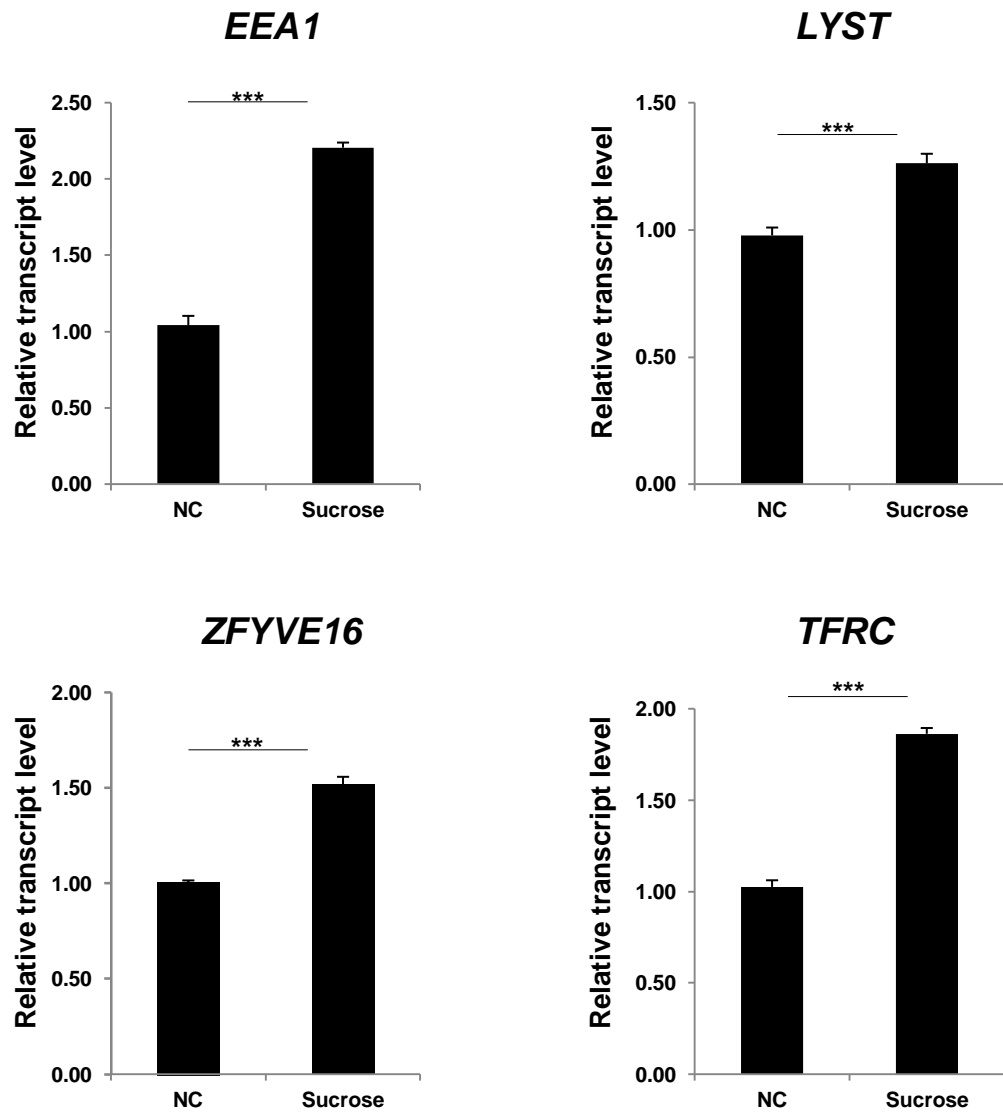**B**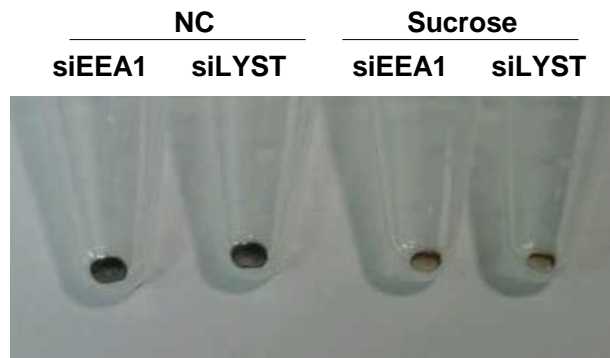**Figure S2.**

**A**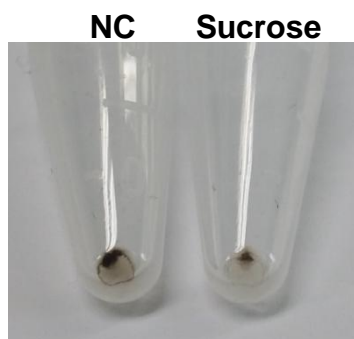**B**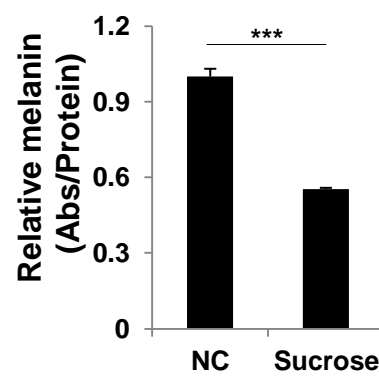**C**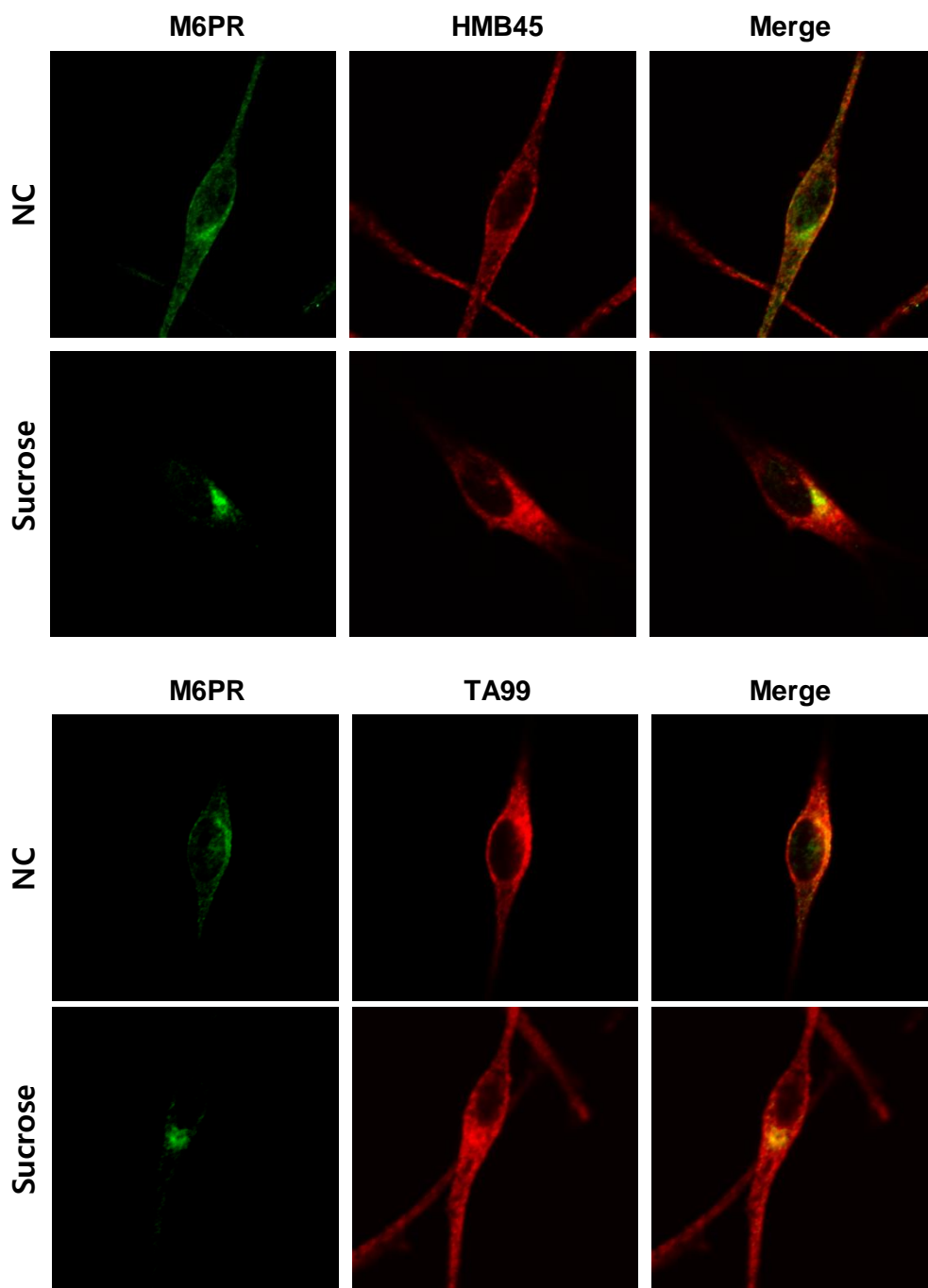

Figure S3.

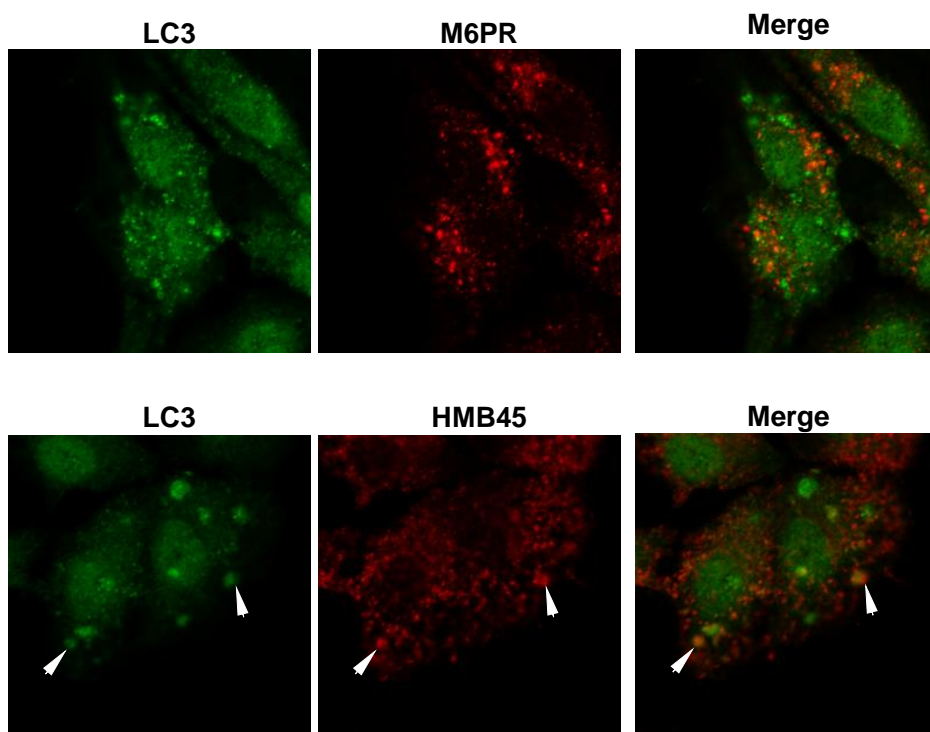

Figure S4.

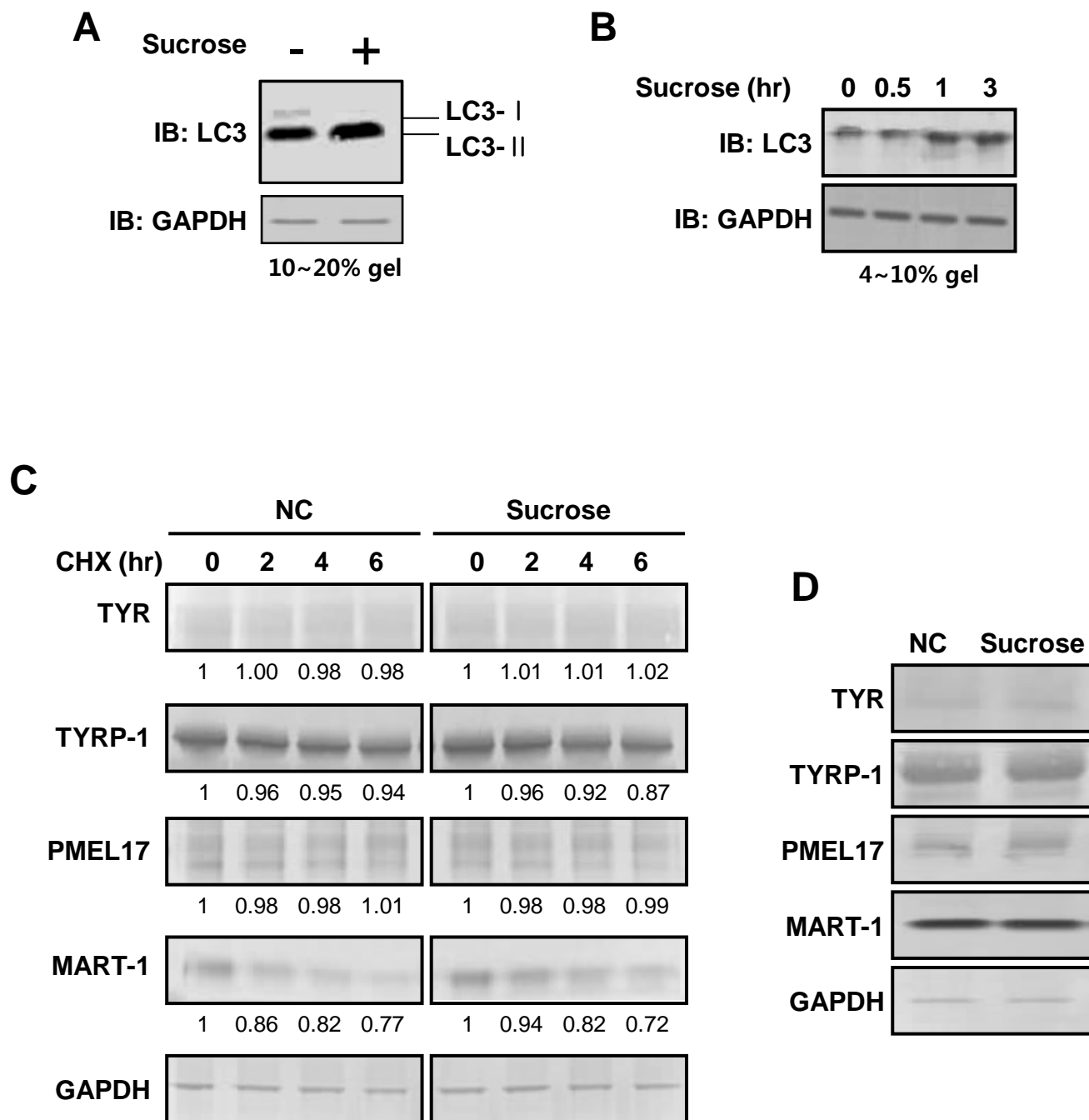

Figure S5.

**A**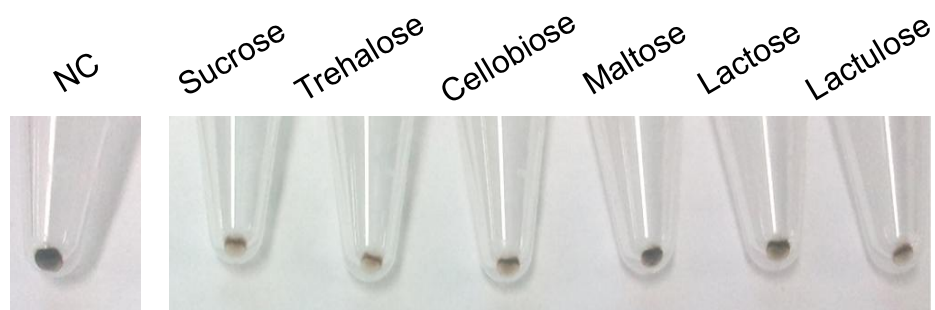**B**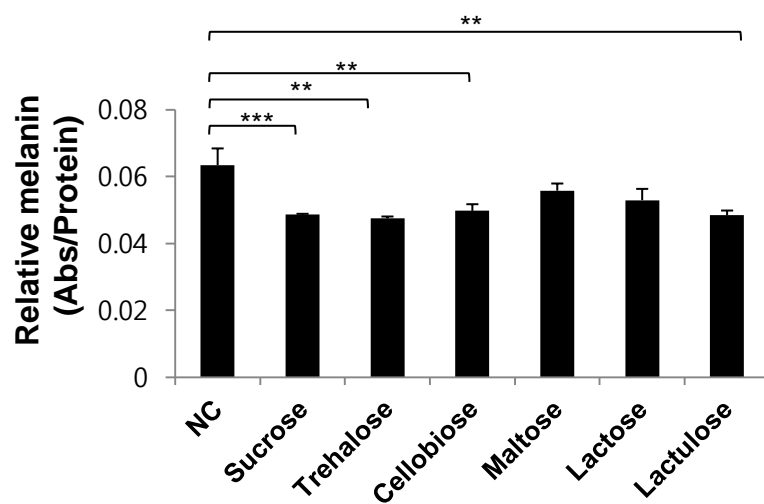**Figure S6.**

Supplement: File S1 — Figure S1. The expression and processing of melanogenesis-related proteins is normal under hyperosmotic stress. (A) MNT-1 cells were treated with 50 mM sucrose for 7 days, and the expression levels of melanogenesis-related proteins were analyzed by western blotting using the indicated antibody. NC, negative control. (B) The recognition sites of PMEL17 by aN or an anti-HMB45 antibody are schematically represented. aN, anti-PMEL17 antibody that recognizes the N-terminal region of PMEL17, as indicated. (C) The processing of PMEL17 in MNT-1 cells treated with 50 mM sucrose for 7 days was analyzed by western blotting. NC, negative control. Figure S2. The expression of vesicle transport-related transcripts. (A) Non-treated (NC) or MNT-1 cells treated with 50 mM sucrose for 7 days were harvested. The expression levels of vesicle transport-related transcripts that were up-regulated under hyperosmotic stress in the microarray data (Table S1) were quantified using RT-qPCR. The data are representative of three independent experiments (***, P < 0.005). (B) Each siRNA was treated twice every third day during sucrose treatment for 7 days, and the color of the cell pellets was monitored. NC, negative control. Figure S3. Hyperosmotic stress down-regulates melanin production in normal human melanocytes by inducing abnormal, swollen melanosomes. (A) Normal human melanocytes were treated with 50 mM for 7 days, and the color of the cell pellets was monitored. NC, negative control. (B) The melanin content was measured at 450 nm. The data are representative of three independent experiments (***, P < 0.005). (C) Hyperosmotic stress-induced M6PR-positive swollen vacuoles contain TYRP-1 and PMEL17 in normal human melanocytes. Cells were treated with 50 mM sucrose for 24 hours and stained with anti-TA99 or anti-HMB45 antibodies. The fluorescence images were acquired using confocal microscopy at a magnification of 1260×. NC, negative control. Figure S4. Hyperosmotic stress induces LC3-positiv [file pone.0105965.s001.pdf]
