# Supplementary material for: Hyperosmotic Stress Reduces Melanin Production by Altering Melanosome Formation
Source: PLoS One. 2014 Aug 29;9(8):e105965. doi: 10.1371/journal.pone.0105965 (PMC4149489; doi:10.1371/journal.pone.0105965)
Supplement: Table S1 — List of up- or down-regulated genes under hyperosmotic stress. The raw intensity values (before and after sucrose treatment), log2-fold-changes, and adjusted P-values (Pad) computed from two-tailed t-tests and median ratio tests (see Materials and Methods) are given. The up- or down-regulated genes are represented by each ‘up’ or ‘down’ in the table. (PDF) [file pone.0105965.s002.pdf]

Table S1. List of up- and down-regualted genes under hyperosmotic stress

Identity			Raw data						
ProbeName	EntrezID	Symbol	- sucrose_1	- sucrose_2	+ sucrose_1	+ sucrose_2	log2-fold-changes	Pad	DEG
A_23_P136909	79366	HMGN5	1170	900	2789	2511	1.514	1.428E-04	up
A_23_P136909	79366	HMGN5	1308.5	1125.5	3139	2814.5	1.447	1.464E-04	up
A_23_P136909	79366	HMGN5	1225	1092.5	2914	2717	1.433	1.060E-04	up
A_23_P136909	79366	HMGN5	1393	1169.5	3226	2910	1.416	1.573E-04	up
A_23_P136909	79366	HMGN5	1014.5	840	2330.5	2054.5	1.388	2.442E-04	up
A_23_P136909	79366	HMGN5	1160	933	2500	2395.5	1.380	1.439E-04	up
A_23_P136909	79366	HMGN5	1155	969	2647	2337	1.377	2.418E-04	up
A_23_P136909	79366	HMGN5	1147	1007.5	2582	2349	1.342	2.119E-04	up
A_23_P136909	79366	HMGN5	1136.5	992	2527.5	2299	1.328	2.169E-04	up
A_23_P136909	79366	HMGN5	1073	970	2395	2227	1.324	1.764E-04	up
A_24_P825874	445582	POTEE	34	34	73	85	1.250	1.604E-05	up
A_23_P67896	6328	SCN3A	107	64	161	212	1.244	8.910E-04	up
A_23_P110791	1436	CSF1R	51	49	81	163	1.243	1.638E-03	up
A_23_P154411	9360	PPIG	1475	1236	2567	2379	1.020	6.143E-04	up
A_24_P310630	65109	UPF3B	810	717	1305	1487.5	1.015	1.949E-05	up
A_33_P3291569	51747	LUC7L3	12188	10414.5	21026	20270	1.013	2.680E-04	up
A_23_P133338	54825	CDHR2	77	52	103	133.5	0.955	1.596E-03	up
A_23_P254573	27000	DNAJC2	7971.5	6583	12731	12379.5	0.937	4.342E-04	up
A_23_P49448	79152	FA2H	294	247	479	468.5	0.930	6.837E-04	up
A_33_P3236698	138649	ANKRD19P	436	385	695	728	0.924	2.544E-04	up
A_32_P185637	57642	COL20A1	991	860	1382	1735	0.892	1.374E-04	up
A_24_P941787	8899	PRPF4B	2422.5	1921	3596.5	3566.5	0.883	7.517E-04	up
A_33_P3514859	100506342	LOC100506342	189	158	225	388	0.880	3.243E-03	up
A_32_P16315	347688	TUBB8	396	349	598	656	0.878	1.377E-04	up
A_23_P39814	9541	CIR1	1969	1531	2903	2813.5	0.870	1.084E-03	up
A_33_P3295358	51129	ANGPTL4	88	77.5	132	154.5	0.859	4.007E-05	up
A_23_P52986	220001	VWCE	861	720.5	1260	1281	0.835	6.256E-04	up
A_33_P3263432	8515	ITGA10	204	167	282	326.5	0.820	2.713E-04	up
A_33_P3256282	386679	KRTAP10-2	82	63	109	134	0.815	9.384E-04	up
A_33_P3374443	3897	L1CAM	91	71	119	150.5	0.806	8.659E-04	up
A_23_P91512	23562	CLDN14	217.5	191	320	341	0.799	3.769E-04	up
A_23_P122615	25957	PNISR	584	504.5	875	834	0.791	1.415E-03	up
A_23_P122615	25957	PNISR	471.5	403	709.5	663	0.787	1.686E-03	up
A_32_P76853	653075	LOC653075	547	478	739	855.5	0.770	4.790E-05	up
A_23_P36865	80184	CEP290	700	588	1035	952	0.766	2.200E-03	up
A_23_P164436	443	ASPA	931	797	1276	1369	0.763	3.588E-04	up
A_33_P3246997	51747	LUC7L3	353	330	526	538.5	0.762	7.976E-04	up
A_23_P76159	8411	EEA1	2552	2266.5	3594	3742	0.761	4.126E-04	up
A_23_P122615	25957	PNISR	545	437	774	732	0.759	2.186E-03	up
A_33_P3345643	65980	BRD9	18305.5	17269	25871	29182	0.756	2.375E-04	up
A_33_P3216232	9270	ITGB1BP1	3852	3162	5208	5330	0.751	7.596E-04	up
A_23_P122615	25957	PNISR	440	378	662	591.5	0.748	2.939E-03	up
A_23_P31224	29992	PILRA	732	703	1004.5	1179	0.747	2.448E-04	up
A_24_P153456	79844	ZDHHC11	9784	8579	13209	14857	0.745	2.133E-05	up
A_23_P122615	25957	PNISR	565	474.5	829	749	0.741	2.856E-03	up
A_23_P122615	25957	PNISR	601.5	523.5	870	834	0.740	1.678E-03	up
A_33_P3368193	119548	PNLIPRP3	225	207	321	349	0.740	3.344E-04	up
A_33_P3364373	440021	KRTAP5-2	310	265	461.5	420	0.737	2.626E-03	up
A_32_P148824	54953	C1orf27	636	541.5	923.5	852.5	0.732	2.424E-03	up
A_33_P3271316	54913	RPP25	174	120.5	188	271	0.730	4.275E-03	up
A_23_P368779	163071	ZNF114	272.5	263.5	381.5	439	0.728	3.137E-04	up
A_33_P3284036	23562	CLDN14	197	176	281	292.5	0.726	6.679E-04	up
A_33_P3423551	8870	IER3	1809	1788	2483.5	2871	0.721	5.001E-04	up
A_33_P3395952	57642	COL20A1	297.5	263.5	398.5	455	0.721	8.879E-05	up
A_23_P13548	25884	CHRDL2	67	51	88	96	0.714	1.657E-03	up
A_23_P65262	10443	N4BP2L2	594.5	502.5	735.5	906	0.713	2.838E-04	up
A_24_P403244	29990	PILRB	3059	2694	3864	4576	0.708	6.943E-05	up
A_33_P3718269	285628	LOC285628	3789	3145	5020.5	5081.5	0.704	1.099E-03	up
A_23_P253524	1062	CENPE	2121	1704.5	2655	2907	0.702	6.979E-04	up
A_33_P3503279	284257	BOD1P	67	51.5	81.5	104	0.702	2.128E-03	up
A_23_P309261	10142	AKAP9	2843	2282	3587	3868	0.701	7.281E-04	up
A_33_P3329834	55119	PRPF38B	955	794	1278	1278	0.701	1.408E-03	up
A_23_P35564	25956	SEC31B	929.5	797	1232	1299	0.700	6.155E-04	up
A_23_P122615	25957	PNISR	523	455	738	709	0.699	1.990E-03	up
A_23_P253524	1062	CENPE	2331	1949.5	3028.5	3187	0.695	6.599E-04	up
A_33_P3327165	343099	CCDC18	290.5	249	399.5	403	0.694	1.264E-03	up
A_24_P332230	51574	LARP7	1433	1161	1971	1791	0.692	3.461E-03	up
A_23_P254434	10739	RFPL2	349	299.5	459	502	0.690	4.287E-04	up
A_33_P3407469	57644	MYH7B	1184	1041	1468	1778	0.690	1.276E-04	up
A_23_P253524	1062	CENPE	2200	1817	2807	2993	0.687	6.407E-04	up
A_24_P179183	23253	ANKRD12	568	491.5	773	774.5	0.686	1.481E-03	up
A_23_P154025	6741	SSB	9216	8201.5	12577	12820	0.683	5.713E-04	up

A_23_P26094	79811	SLTM	1385	1128.5	1854	1778	0.682	2.296E-03	up
A_24_P380679	286006	C7orf53	307	246	413	401.5	0.682	2.543E-03	up
A_23_P253524	1062	CENPE	2041	1726.5	2558	2866	0.680	2.586E-04	up
A_23_P122615	25957	PNISR	504	434	684	676	0.672	1.748E-03	up
A_23_P122615	25957	PNISR	396.5	365	563	547	0.670	2.196E-03	up
A_23_P115842	55749	CCAR1	2322.5	1973	3016	3120	0.670	8.261E-04	up
A_23_P253524	1062	CENPE	1841.5	1565.5	2326.5	2553	0.670	3.669E-04	up
A_23_P253524	1062	CENPE	1560	1468.5	2072.5	2279	0.669	4.259E-04	up
A_33_P3280157	727708	SNORD116-19	1621.5	1306	1952	2231	0.669	6.411E-04	up
A_23_P48676	5836	PYGL	475	409	596	681	0.668	2.049E-04	up
A_23_P250404	10111	RAD50	1148	994	1527	1543	0.668	1.290E-03	up
A_23_P253524	1062	CENPE	1805	1585.5	2314	2524	0.662	2.935E-04	up
A_33_P3270369	100128342	LOC100128342	77	56	91	108.5	0.658	3.075E-03	up
A_23_P160318	1307	COL16A1	109.5	89	146	149.5	0.658	1.579E-03	up
A_33_P3226407	389634	LOC389634	538	510	716.5	793	0.658	5.035E-04	up
A_33_P3278826	4058	LTK	149	124.5	172	236.5	0.657	1.999E-03	up
A_23_P250404	10111	RAD50	1330	1088	1717	1699	0.655	2.021E-03	up
A_33_P3263538	283131	NEAT1	601	515.5	712	889	0.651	4.339E-04	up
A_23_P99762	58517	RBM25	3410	2963	3761	5283.5	0.650	2.428E-03	up
A_23_P100189	5619	PRM1	75	55	88	106	0.650	3.023E-03	up
A_32_P89827	374491	LOC374491	247	215	332	339.5	0.650	1.386E-03	up
A_32_P35512	6741	SSB	12379	8704	14918.5	14797	0.648	3.640E-03	up
A_23_P5586	10199	MPHOSPH10	9247.5	7348.5	11644	11895	0.648	1.338E-03	up
A_24_P286054	9765	ZFYVE16	2867	2328.5	3604	3653	0.644	1.642E-03	up
A_23_P250404	10111	RAD50	1384	1140.5	1740.5	1807	0.643	1.352E-03	up
A_33_P3327956	100289635	ZNF605	812	671.5	1034	1056.5	0.643	1.698E-03	up
A_23_P168771	57639	CCDC146	190	166	243	275	0.643	2.034E-04	up
A_33_P3421490	23251	KIAA1024	1659	1460.5	2283.5	2096	0.638	3.672E-03	up
A_33_P3294524	23253	ANKRD12	191	190	266.5	286	0.638	1.306E-03	up
A_32_P189781	645687	C14orf34	355	299	444	490	0.636	6.574E-04	up
A_33_P3420386	2916	GRM6	35	33	45	60	0.635	2.563E-03	up
A_23_P253524	1062	CENPE	2146	1795	2688.5	2804	0.634	1.098E-03	up
A_33_P3344204	79844	ZDHHC11	11598.5	11372.5	14962.5	17655.5	0.633	1.051E-03	up
A_23_P115842	55749	CCAR1	2084	1810	2663	2765	0.632	9.428E-04	up
A_33_P3365193	278	AMY1C	61	60.5	84	98.5	0.632	5.541E-04	up
A_23_P362694	260436	C4orf7	134	109	173	180	0.632	1.606E-03	up
A_23_P250404	10111	RAD50	1428	1184	1840	1806	0.631	2.348E-03	up
A_23_P100220	80004	ESRP2	125	89	155	154.5	0.629	5.343E-03	up
A_33_P3380211	10142	AKAP9	574	490.5	726.5	770	0.628	9.582E-04	up
A_33_P3397323	126068	ZNF441	1431	1153	1859	1735	0.628	3.937E-03	up
A_33_P3239101	647042	GOLGA6L10	296	258	399	389	0.628	2.593E-03	up
A_23_P115842	55749	CCAR1	2897	2447	3692	3685	0.625	1.717E-03	up
A_33_P3324894	9026	HIP1R	82.5	63	95	120	0.625	3.063E-03	up
A_23_P250404	10111	RAD50	1391	1174	1802	1762	0.624	2.333E-03	up
A_23_P115842	55749	CCAR1	2482	2265	3095.5	3479	0.623	1.725E-04	up
A_33_P3317752	100132476	KRTAP4-7	116	95.5	152	155.5	0.620	1.863E-03	up
A_23_P161156	220929	ZNF438	326.5	285.5	403	465	0.620	1.539E-04	up
A_23_P42257	8870	IER3	5313	4937	6406	7802	0.620	5.589E-04	up
A_23_P122615	25957	PNISR	431	362	562	549	0.620	3.020E-03	up
A_23_P251412	10590	SCGN	52.5	49	80.5	71	0.619	4.452E-03	up
A_33_P3321781	645460	FLJ44342	1984	1619.5	2495.5	2452	0.613	2.789E-03	up
A_24_P112160	80761	UPK3B	124	112	163	176	0.608	5.354E-04	up
A_23_P154065	7277	TUBA4A	401	364	493.5	579	0.606	4.772E-05	up
A_33_P3313796	91057	CCDC34	3050	2421.5	3901	3527.5	0.606	5.481E-03	up
A_23_P372467	130872	AHSA2	3216	2743	3815	4276	0.606	2.324E-04	up
A_23_P253524	1062	CENPE	1632	1340	1959	2106	0.605	1.094E-03	up
A_33_P3424222	3119	HLA-DQB1	117	103	155.5	161	0.604	1.291E-03	up
A_33_P3668839	644656	LOC644656	371.5	307	485.5	457.5	0.602	4.542E-03	up
A_23_P253524	1062	CENPE	2007	1586.5	2336	2559	0.602	1.579E-03	up
A_23_P115842	55749	CCAR1	2354.5	2078	2948	3102.5	0.601	7.720E-04	up
A_23_P27180	84081	NSRP1	2281	1986	2758	3069	0.600	2.174E-04	up
A_23_P167818	79632	FAM184A	308.5	263	406.5	389.5	0.599	3.714E-03	up
A_23_P115842	55749	CCAR1	2821.5	2453	3534	3641	0.598	1.154E-03	up
A_24_P191833	140890	SREK1	1218.5	1045	1512	1563.5	0.597	1.430E-03	up
A_23_P354074	1130	LYST	651	548	862	780.5	0.597	5.793E-03	up
A_23_P74778	79630	C1orf54	1207	944	1472	1448.5	0.596	3.835E-03	up
A_23_P115842	55749	CCAR1	2580	2186.5	3158	3303	0.596	1.097E-03	up
A_23_P165707	129530	LYG1	86	76	112	121.5	0.595	4.453E-04	up
A_23_P250404	10111	RAD50	1079.5	944	1384.5	1369.5	0.595	2.354E-03	up
A_23_P115842	55749	CCAR1	2138.5	1940.5	2764	2786	0.593	1.733E-03	up
A_23_P79360	115677	NOSTRIN	112	87.5	140	143	0.591	3.260E-03	up
A_33_P3330468	55285	RBM41	1814	1618.5	2276.5	2381	0.590	1.115E-03	up
A_33_P3249743	255180	FLJ38723	188.5	175	234	276	0.588	1.665E-04	up
A_23_P312565	138649	ANKRD19P	169.5	158	212	248	0.585	1.585E-04	up
A_23_P107116	7732	RNF112	112	95	138	156.5	0.585	4.994E-04	up
A_23_P212617	7037	TFRC	24681	21102	28959.5	33062.5	0.584	1.806E-04	up
A_23_P126844	8718	TNFRSF25	500	433	614	658	0.583	1.030E-03	up
A_33_P3412468	9481	SLC25A27	201	168.5	259	254	0.582	3.285E-03	up

A_33_P3286754	23541	SEC14L2	630	520	745	814.5	0.582	1.192E-03	up
A_33_P3214061	729176	LOC729176	248	141.5	29.5	33.5	-2.532	2.443E-05	down
A_23_P47340	57453	DSCAML1	145.5	77	27	28	-1.913	1.171E-04	down
A_33_P3294985	554203	JPX	216	95	44.5	50	-1.547	8.632E-04	down
A_23_P166027	23626	SPO11	111	103	39.5	39	-1.398	3.007E-05	down
A_23_P121614	4589	MUC7	1480.5	1390.5	521	819.5	-0.996	1.101E-03	down
A_23_P121614	4589	MUC7	1811	1679	638	994.5	-0.986	9.738E-04	down
A_23_P121614	4589	MUC7	1285	1129	446.5	697	-0.968	1.137E-03	down
A_23_P121614	4589	MUC7	1266.5	1208	449.5	736.5	-0.964	1.627E-03	down
A_23_P121614	4589	MUC7	1428	1324	505	810	-0.963	1.429E-03	down
A_23_P121614	4589	MUC7	1567	1458	563	877	-0.959	1.175E-03	down
A_23_P121614	4589	MUC7	1553	1378	561	836.5	-0.951	8.495E-04	down
A_23_P121614	4589	MUC7	1213	1162	454	688.5	-0.945	1.139E-03	down
A_23_P121614	4589	MUC7	1527.5	1409	578	853.5	-0.918	9.331E-04	down
A_23_P121614	4589	MUC7	1668	1636	665	995	-0.876	1.369E-03	down
A_33_P3421867	266727	MDGA1	488	385	207	276	-0.746	1.256E-03	down
A_23_P4400	653240	KRTAP4-11	417	434	208	268	-0.741	1.213E-03	down
A_23_P111995	4017	LOXL2	315.5	296.5	154	198	-0.708	6.271E-04	down
A_23_P111995	4017	LOXL2	279	274	146.5	176.5	-0.683	5.757E-04	down
A_23_P111995	4017	LOXL2	319	288	156	206	-0.660	9.667E-04	down
A_23_P111995	4017	LOXL2	306.5	275	156	189.5	-0.653	1.804E-04	down
A_32_P100683	23331	TTC28	574	518	282.5	371.5	-0.629	9.648E-04	down
A_23_P111995	4017	LOXL2	244	213	132	147	-0.619	2.412E-04	down
A_23_P82523	5243	ABCB1	459	455	237	323	-0.611	2.441E-03	down
A_23_P90273	64377	CHST8	1180.5	1055	593	739	-0.609	3.029E-04	down
A_23_P111995	4017	LOXL2	334	300	180	210	-0.601	3.667E-05	down
A_23_P111995	4017	LOXL2	283	251	150.5	179	-0.599	1.311E-04	down
A_23_P49145	653808	ZG16	72.5	55	40	42.5	-0.583	3.146E-03	down
A_33_P3406828	100132288	TEKT4P2	342.5	239	170	186.5	-0.582	5.653E-03	down
